# UNAIDS 95-95-95 targets in older people living with HIV in urban and rural KwaZulu-Natal, South Africa

**DOI:** 10.1186/s12879-026-13273-y

**Published:** 2026-04-07

**Authors:** Etheldreda I. Y. Madela, Celia L. Gregson, Farhanah Paruk, Anya J. Burton, Rita Patel, Hannah Wilson, Fundile Habana, Anthony M. Manyara, Bavumile Mbanjwa, Lucy Gates, Camille M. Pearse, Chris Grundy, Kate A. Ward, Bilkish Cassim

**Affiliations:** 1 Department of Geriatrics, University of KwaZulu-Natal, KwaZulu- Natal, Durban, South Africa; 2 Global Health and Ageing Research Unit, Bristol Medical School, University of Bristol, Bristol, UK; 3 Division of Internal Medicine, University of KwaZulu-Natal, KwaZulu-Natal, Durban, South Africa; 4 Musculoskeletal Research Unit, Translational Health Sciences, Bristol Medical School, University of Bristol, Bristol, UK; 5 Department of Rheumatology, University of KwaZulu-Natal, KwaZulu-Natal, Durban, South Africa; 6 MRC Lifecourse Epidemiology Centre, University of Southampton, Southampton, England, UK; 7 MRC International Statistics and Epidemiology Group, London School of Hygiene and Tropical Medicine, London, UK

**Keywords:** HIV, Older persons, South Africa, UNAIDS 95-95-95 targets, Viral load

## Abstract

**Background:**

Data describing HIV prevalence, ART use and virological suppression, in older adults in urban or rural South Africa, are limited. We aimed to address this evidence gap.

**Methods:**

In a population-based cross-sectional study, using age- and sex-stratified random sampling of adults aged ≥ 40 years, a researcher-administered questionnaire collected socio-demographic, and clinical data (03/2022-04/2024). HIV was confirmed using two point-of-care tests (discrepancies resolved by ELISA). The age- and sex-specific study prevalence was applied to the KwaZulu Natal (KZN) province population structure to provide an illustrative projection of HIV prevalence in KZN. Achievement of UNAIDS 95-95-95 targets was calculated in 10-year age bands. People living with HIV (PLHIV) were categorised as virologically suppressed (< 50copies/mL) vs. unsuppressed, and younger (40-49years) vs. older (≥ 50years). Logistic regression determined associations with HIV and virological suppression.

**Results:**

1,916 adults were recruited; 713 (37.2%) living with HIV, 36.7% men and 37.7% women. HIV prevalence was 68.1% among 40–49-year-olds, 49.4% among 50–59-year-olds, 26.0% among 60–69-year-olds and 9.8% age ≥ 70 years. In older adults (≥ 50 years), the prevalence was 27.9%. The overall projected standardised HIV prevalence in adults ≥ 40 years in KZN was 47.4% (44.8% men; 49.3% women). In our sample, 98% of PLHIV were aware of their status, 97.6% on ART, and 77.7% virologically suppressed. Men ≥ 70 years achieved highest virological suppression (88.2%). Overall, being female vs. male (57.8% vs. 42.2%; OR 1.6 [95%CI 1.1, 2.4]; *p* = 0.008), having HIV ≥ 3 years vs. < 3 years (62.2% vs. 55.1%; OR 3.0 [95%CI 1.6, 5.7]; *p* = 0.001), rural vs. urban living (50.9% vs. 49.1%; OR 1.5 [95%CI 1.0, 2.1]; *p* = 0.044) were associated with virological suppression. Older PLHIV (≥ 50years) vs. younger (40-49years) reported hypertension (51.0% vs. 30.0%), diabetes (10.0% vs. 5.0%), and polypharmacy (≥ 5 drugs) (13.2% vs. 6.3%).

**Conclusion:**

In KZN, the overall study prevalence of HIV in adults age ≥ 50 years was 27.9% in both urban and rural populations, the UNAIDS 95-95-95 targets were met for known status and being on treatment, but not virological suppression.

**Supplementary Information:**

The online version contains supplementary material available at 10.1186/s12879-026-13273-y.

## Introduction

The world’s older population (age 60 years and above) is increasing and is projected to surpass the population aged under five years by 2050 [1]. Overall life expectancy in South Africa (SA) has increased from 56.3 years to 61.1 years [2]. Of the estimated 39.9 million people living with HIV (PLHIV) globally, 7.7 million live in SA, with the highest prevalence in the province of KwaZulu-Natal (KZN), where 1.9 million PLHIV reside [3, 4]. The availability of effective antiretroviral therapy (ART) has led to a 57% reduction in acquired immunodeficiency syndrome (AIDS) related deaths in sub-Saharan Africa (SSA), including SA, between 2010 and 2023 [3]. The life expectancy of PLHIV is therefore expected to increase in the region.

Studies in PLHIV have focused largely on younger adults (15–49 years) and only recently has the prevalence of HIV in people over 50 years been reported [5], with even fewer reports in persons aged ≥ 65 years. In 2012, the Joint United Nations Programme on HIV/AIDS (UNAIDS) estimated that of the 3.6 million people aged ≥ 50 years living with HIV (LHIV) the majority (estimated 2.9 million) resided in low- and middle-income countries [5]. In SA, the reported prevalence of HIV in adults aged ≥ 50 years ranges between 13 and 18% [6–8], which is similar to Swaziland (13%) [5], but higher than Ethiopia (6.5%) [9], and Kenya (5%) [5–9].

The UNAIDS 90-90-90 targets, instituted in 2013, were to be achieved by 2020 [10]. The aim was for 90% of PLHIV worldwide to know their status, 90% of those to receive ART and 90% of those on ART to be virologically suppressed. These targets have since been increased to 95-95-95 to be achieved by 2025 [3]. A population-based survey conducted in 2017 reported UNAIDS 90-90-90 targets to be 85%-71%-88%, respectively in PLHIV aged 15 to 64 years across all provinces in both urban and rural SA [11] while 95-95-95 by the March 2024 Department of Health South African National AIDS council (SANAC) report in March 2025 was (95-79-74) in the total population [12]. The 95-95-95 targets for Zimbabwe in adults aged ≥ 40 years were 93-96-90 [13]. Comparable data on the 95-95-95 targets are needed for SA to assess success for the ART rollout programme in KZN.

South Africa has experienced several policy changes in the management of the HIV pandemic. In April 2004, ART was made available for the first time in the public sector to PLHIV with a CD4 cell count ≤ 200 cells/µL. Subsequently, the CD4 cell count threshold increased to ≤ 350 cells/µL, but only for pregnant women and patients with tuberculosis (TB). In 2012, pregnant women and patients with TB were eligible for treatment regardless of their CD4 cell count and the threshold increased to CD4 ≤ 350 cells/µL for all other PLHIV. The Universal Test and Treat guidelines were implemented in September 2016 to expand the benefits of ART to all PLHIV, and in 2023 all PLHIV were eligible to start ART regardless of the CD4 cell counts. The initiation of ART on the same day or within seven days of diagnosis in those with no contraindications aimed to ensure early initiation and better virological suppression [11, 14–20].

Ageing is associated with increased risk of non-communicable diseases (NCDs), especially cardiovascular diseases, multimorbidity and polypharmacy. HIV infection is thought to accelerate ageing [21, 22], increasing risk of age-associated conditions, making older PLHIV more susceptible to NCDs [8, 23–26]. There is a paucity of up-to-date data on the prevalence and clinical profiles of older PLHIV in diverse regions of SA. The purpose of this study is to determine HIV prevalence in persons aged 40 years and older, and describe their sociodemographic and clinical features, and the achievement of UNAIDS (95-95-95) targets in urban and rural KZN.

## Methods

### Study population and recruitment

A population-based cross-sectional study was conducted in KwaMashu, a large urban area 12 km (km) north of eThekwini, and a rural area, Mafakathini, in the uMgungundlovu district, located approximately 100 km south west from eThekwini. Data were collected between 8 March 2022 and 12 April 2024 as part of the Fractures-E3 Study (Fractures in sub-Saharan Africa: epidemiology, economic impact, and ethnography). Satellite images and OpenStreetMap, available through Aeronautical Reconnaissance Coverage Geographic Information System (ARCGIS) software, were used to map both areas in detail. A set of random points were selected within each area. Around these random locations, road-based ‘blocks’ were selected, of a size suitable to recruit approximately three eligible individuals in each stratum (3 male and 3 females in each age group of (40–54 years, 55–69 years and ≥ 70 years). Blocks were accessed following their random order, and households within a block approached sequentially until the recruitment quota was fulfilled. If there was no one at the household, fieldworkers returned twice before marking the property as vacant. If there were insufficient eligible individuals within the block the team moved on to the next block. Population denominator data by sex and five-year age-bands, obtained through national census data (2011), were used to build a stratified random quota sampling frame. The study aimed to recruit 168 men and 168 women in each of three age strata 40–54 years, 55–69 years and ≥ 70 years to reach a total of 504 women and 504 men from each site. Adults were eligible as residents if they had lived and shared meals in the household for at least four weeks and were able to give written or thumbprint consent. All consent was taken in isiZulu. Legal guardian (proxy) written/ thumbprint consent was sought for those who lacked capacity to consent due to cognitive impairment. Pregnant women were excluded as the original study included radiography. Consenting participants were assessed at the local clinic (see protocol paper of Fractures-E3: epidemiology, economic impact and ethnography of fragility fractures study) in which this study was embedded [27].

### Data collection and measurements

Data were collected using electronic questionnaires (Supplement Table 1) and standardized examination forms and entered directly into Research Electronic Data Capture (REDCap)^®^ [28], with inbuilt data validation checks. Sociodemographic factors, marital status, level of education, medical history, all current medicines, smoking and alcohol consumption were recorded. A wealth index was derived by Principal Component Analysis combining house ownership, housing characteristics (roof and wall material) and household assets ownership (e.g. television, refrigerator, car) to derive wealth tertiles (low, middle, and high) [29]. Food insufficiency in the last four weeks was determined using five *Yes/No* questions taken from the Household Food Insecurity Access Scale [30].

In the standardised examination, all measurements were performed by trained research staff, following standardised operating procedures. Weight was measured in kilograms (kg) using a calibrated Seca^®^ scale (participants wearing minimal clothing, shoes and socks removed, pockets emptied). Standing height was measured in millimetres (mm) using a portable Seca^®^ stadiometer (shoes, socks, hats, head scarves removed). Mid-upper arm circumference (MUAC) was measured on the non-dominant arm using an ergonomic Seca^®^ measuring tape; <24 centimetres (cm) was considered low [31]. All measurements were repeated and the mean calculated. If there was a difference of > 0.5 kg or >5 mm between respective measurements, two additional measurement was taken and a mean of the two new measurements was used. Body mass index (BMI) (kg/m^2^) was calculated by dividing weight (kg) by height (metres) squared. World Health Organization (WHO) categories were used to classify BMI as underweight: <18.5 kg/m^2^, normal: 18.5–24.9 kg/m^2^, overweight: 25–29.9 kg/m^2^, obese class I: 30–34.9 kg/m^2^, obesity class II: 35–39.9 kg/m^2^ and morbidly obese (class III): ≥40 kg/m^2^.

### HIV definition

Living with HIV was self-reported based on a previous positive result, being on ART, or a positive diagnosis at the research clinic on two rapid point-of-care (Alere Determine^®^ [Alere North America, USA] and Chembio HIV 1/2 STAT-PAK^®^ [Chembio Diagnostic Systems, Inc, Medford, New York, USA]) tests, or an ELISA test if the point-of-care tests were discordant. All participants who reported being negative or not knowing their status were offered the tests. In PLHIV, 224 had viral load (VL) results taken within the previous year available (these were retrieved from the National Health Laboratory Services (NHLS) database. Blood was collected for VL testing in a further 413, and processed by Lancet laboratory using the ABBOTT RealTime HIV-1 Assay. Virological suppression was defined as a VL < 50 copies/mL (15).

### Comorbid diseases and polypharmacy

Comorbid diseases were self-reported based on a previous diagnosis by a health professional, use of medication for a disease, or a new diagnosis made at the study clinic. Previous history of TB was self-reported. A random blood glucose of ≥ 11.1mmol/l or fasting blood glucose of ≥ 7, and blood pressure ≥ 140/90mmHg on two or more measurements at the clinic were diagnostic of diabetes mellitus and hypertension, respectively. Depression and anxiety were identified using the Shona Symptom Questionnaire (SSQ); a score of 8–14 or red flags (auditory or visual hallucinations or suicidal ideation), were regarded as positive for mental health disorder [28]. Polypharmacy was defined as use of five or more drugs/day. These included prescribed and over the counter drugs, but excluded topicals, supplements and vitamins (except for iron, thiamine, and vitamin D). The justification for including thiamine, iron and vitamin D stems from these drugs being prescribed for medical conditions. Each ART was counted as one drug.

### Ethical and governance approvals

Ethical approval was obtained from the University of KwaZulu-Natal’s Biomedical Research Ethics Committee reference numbers BREC/00002513/2021 and BREC/00005591/2023. Permission to conduct the study was granted by the Health Committee of the local area councils and the District and Provincial Departments of Health (NHRD Ref: KZ_202106_024).

### Statistical analysis

Analyses were performed using Stata^®^ SE version 17 [32]. Data were assessed for normality using histograms. Participant characteristics were summarised using descriptive statistics; continuous data were summarised using means with standard deviation (SD) if normally distributed, or median with interquartile range (IQR) if not normally distributed. Categorical values were summarised using frequency counts and percentages. Descriptive statistics of demographic and clinical variables were compared between PLHIV and participants who were HIV negative. All analyses were adjusted for age.

The prevalence of HIV was calculated for the study population overall and stratified by sex, and 10-year age band, with confidence intervals calculated using the Wald method. Assuming study prevalence estimates were generalizable to the KZN population, the age- and sex-specific study prevalence estimates were applied to the KZN population structure (Statistics SA Census 2022), i.e. total HIV prevalence in people age ≥ 40 years was projected by multiplying the proportion ≥ 40 years in the KZN population in each strata by the strata-specific prevalence, and adding these together.

The 95-95-95 targets were calculated according to UNAIDS guidelines [3].

Participants LHIV were stratified by virological suppression and by age (40–49 vs. ≥50 years). Confidence intervals were calculated using the Wald method, or the Wilcon method for proportions close to 0 or 1. Logistic regression determined associations by HIV status, and virological suppression. Models included complete cases only. The Odds Ratios (OR) and 95% Confidence Intervals (95% CI) for association by HIV status and virological suppression were adjusted for age. A model fit was assessed considering age as either categorical or continuous variable and compared models using the Likelihood ratio test. Age as a continuous variable had a lower Akaike Information Criterion (AIC) and was therefore used when adjusting for age. Factors associated with virological suppression with a p-value < 0.1 in univariate analysis were added to a multivariate stepwise backward selection, where a p-value < 0.05 indicated those independently associated with virological suppression.

## Results

In total, 6,235 households were approached; 2,572 people aged ≥ 40 years were identified of whom 2,134 (83.0%) were invited to participate, and 1,916 (89.8%) consented to participate in the study (Supplementary Fig. 1). All 1,916 participants had HIV status established. The median age was 60 years [IQR 50–70 years] and 53.9% were women (Table 1).

**Table 1:** Sociodemographic and clinical characteristics of the study population and associations with HIV

	Total	HIV Negative	PLHIV	Unadjusted		Adjusted for age	
	n=1916	n=1203 (62.8)	n=713 (37.2)	OR, 95% CI	p value	OR, 95% CI	p value
**Socio-demographics**							
Age in years (median [IQR])	60 [50-70]	65 [57-72]	51 [46-59]	0.9 (0.8-0.9)	< 0.001		
Age groups, n (%)							
40-49 years	445 (23.2)	142 (11.8)	303 (42.5)	reference			
50-59 years	490 (25.6)	248 (20.6)	242 (33.9)	0.5 (0.4-0.6)	< 0.001		
60-69 year	442 (23.1)	327 (27.2)	115 (16.1)	0.2 (0.1-0.2)			
≥ 70 years	539 (28.1)	486 (40.4)	53 (7.4)	0.1 (0.03-0.1)			
Sex, n (%)							
Men	883 (46.1)	559 (46.5)	324 (45.4)	reference		reference	
Women	1034 (53.9)	644 (53.5)	389 (54.6)	1.0 (0.9-1.3)	0.663	1.4 (1.1-1.7)	0.002
Site, n (%)							
Urban	968 (50.5)	618 (51.4)	350 (49.1)	reference		reference	
Rural	948 (49.5)	585 (48.6)	363 (50.9)	1.1 (0.9-1.3)	0.334	1.1 (0.9-1.4)	0.209
Marital status, n (%)							
Never married	921 (48.1)	451 (37.5)	470 (65.9)	reference		reference	
Married/ Cohabitating	605 (31.6)	441 (36.6)	164 (23.0)	0.4 (0.3-0.4)	< 0.001	0.6 (0.5-0.8)	< 0.001
Separated/ divorced	80 (4.2)	49 (4.1)	31 (4.4)	0.6 (0.4-1.0)		1.2 (0.7-2.0)	
Widowed	304 (15.9)	256 (21.3)	48 (6.7)	0.2 (0.1-0.3)		0.7 (0.5-1.0)	
Unknown	6 (0.3)	6 (0.5)	0				
Level of education, n (%)							
None/Primary	743 (38.8)	552 (45.9)	191 (26.8)	reference			
Secondary to Tertiary	1166 (60.9)	645 (53.6)	521 (73.1)	2.3 (1.9-2.9)	< 0.001	0.9 (0.7- 1.2)	0.46
Missing	7 (0.4)	6 (0.5)	1 (0.1)				
Wealth index, n (%)							
High	278 (14.5)	193 (16.0)	85 (11.9)	reference			
Middle	848 (44.3)	561 (46.6)	287 (40.3)	1.2 (0.9-1.6)	< 0.001	1.0 (0.8-1.5)	< 0.001
Low	790 (41.2)	449 (37.3)	341 (47.8)	1.7 (1.3-2.3)		1.4 (1.0-1.9)	
Smoking, n (%)							
Past or never	1438 (75.1)	935 (77.7)	503 (70.6)	reference			
Current smoking	472 (24.6)	263 (21.9)	209 (29.3)	1.5 (1.2-1.8)	< 0.001	0.8 (0.6-1.0)	0.11
Missing	6 (0.3)	5 (0.4)	1 (0.1)				
Alcohol consumption, n (%)							
None	1274 (66.5)	854 (71.0)	420 (58.9)	reference			
Drinks alcohol	631 (32.9)	339 (28.2)	292 (41.0)	1.8 (1.4-2.1)	< 0.001	0.9 (0.7-1.1)	0.414
Missing	11 (0.6)	10 (0.8)	1 (0.1)				
Food insecurity, n (%)							
None to mild (score 0-2)	935 (48.8)	625 (52.0)	310 (43.5)	reference			
Moderate to severe (score 3-5)	977 (51.0)	574 (47.7)	403 (56.5)	1.4 (1.2-1.7)	< 0.001	1.3 (1.0-1.6)	0.025
Missing	4 (0.2)	4 (0.3)					
**Clinical**							
Comorbidity, n (%)							
Hypertension	1095 (57.2)	795 (66.1)	300 (42.1)	0.4 (0.3-0.5)	< 0.001	0.8 (0.6-0.9)	0.024
Diabetes mellitus	281 (14.7)	225 (18.7)	56 (7.9)	0.4 (0.3-0.5)	< 0.001	0.6 (0.4-0.9)	0.004
Previous tuberculosis	218 (11.4)	73 (6.1)	145 (20.3)	3.9 (2.9-5.3)	< 0.001	3.2 (2.3-4.5)	< 0.001
Depression and anxiety	166 (8.7)	87 (7.2)	79 (11.1)	1.6 (1.2-2.2)	0.004	1.0 (0.7-1.4)	0.986
Polypharmacy, n (%)	226 (11.8)	115 (9.6)	111 (15.6)	1.7 (1.3-2.3)	< 0.001	3.4 (2.4-4.7)	< 0.001
MUAC, n (%)							
Normal ( ≥ 24 cm)	1154(60.2)	740 (61.5)	414 (58.0)	reference			
Low (<24 cm)	703 (36.7)	425 (35.3)	278 (39.0)	1.2 (0.9-1.4)	0.113	1.1 (0.9-1.4)	0.322
Missing	59 (3.1)	38 (3.2)	21 (3.0)				
BMI categories (kg/m^2^), n (%)							
Normal: 18.5-24.9	546 (28.5)	305 (25.4)	241 (33.8)	reference		reference	
Underweight: < 18.5	72 (3.8)	39 (3.2)	33 (4.6)	1.1 (0.7-1.8)	0.003	1.0 (0.6-1.7)	< 0.001
Overweight: 25-29.9	412 (21.5)	264 (22.0)	148 (20.8)	0.7 (0.5-0.9)		1.0 (0.8-1.4)	
Obese class I: 30-34.9	362 (18.9)	231 (19.2)	131 (18.4)	0.7 (0.5-0.9)		1.2 (0.8-1.6)	
Obesity class II: 35-39.9	225 (11.7)	154 (12.8)	71 (10.0)	0.6 (0.4-0.8)		0.7 (0.5-1.1)	
Morbidly obese class III: ≥ 40	250 (13.0)	176 ( 14.6)	74 (10.3)	0.5 (0.4-0.7)		0.6 (0.4-0.9)	
Missing	49 (2.6)	34 (2.8)	15 (2.1)				
Years since diagnosis with HIV		N/A					
< 3 years			47 (6.6)				
≥ 3 years			430 (60.3)				
Unknown			236 (33.1)				
Duration on ART(years):median (IQR)			10 [6-14]				
On ART, n (%)			680 (97.6)				
ART drug combinations							
TEE			55 (7.7)				
TLD			416 (58.4)				
ALD			15 (2.1)				
DTG based regimen			76 (10.7)				
Atazanavir/lopinavir/darunavir			12 (1.7)				
Unknown regimen			139 (19.5)				

Abbreviations:  AOR: age adjusted odds ratios, ART: antiretroviral therapy,  BMI: body mass index, cm: centimetre, IQR: interquartile range, MUAC: mid upper arm circumference, OR: Odds ratio

ART combinations: TEE: tenofovir+ emtricitabine+ efavirenz , TLD: tenofovir+ lamivudine+ dolutegravir or tenofovir+ lamivudine with missing 3^rd^ drug, ALD: abacavir+ lamivudine+ dolutegravir, DTG based: on dolutegravir with 2^nd^ and 3^rd^ drug unknown, Second line: on atazanavir or lopinavir or darunavir

Comorbidity: based on self-report, taking medication for the disease and assessed during the study. Polypharmacy: ≥5 drugs/day (ART commonly = 3 drugs/day)

## HIV prevalence

**Fig. 1:**
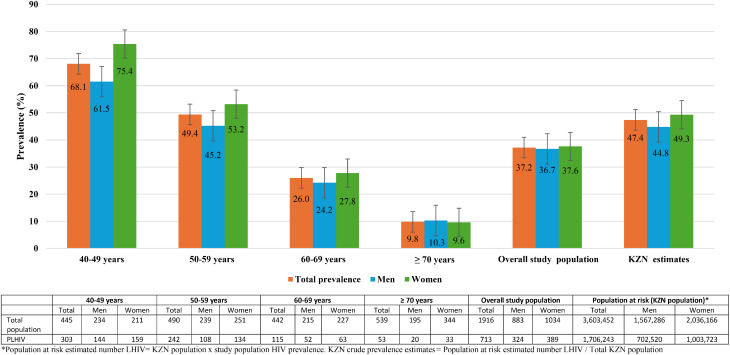
Study and crude KZN HIV prevalence and confidence intervals (95%) stratified by sex and age bands

The overall study prevalence of HIV was 37.2%; 36.7% in men and 37.7% in women, with the highest prevalence (75.4%) in women age between 40 and 49 years and lowest (9.6%) in women age ≥ 70 years (Fig. 1). The overall prevalence in participants age ≥ 50 years was 27.9%. Fourteen participants (0.7%) were newly diagnosed during the study, 5 (35.7%) of whom were ≥ 50 years). When applying the stratified study prevalences to KZN population structure, the projected KZN prevalence for the population aged ≥ 40 years was 47.4%; 44.8% in men and 49.3% in women (Fig. 1).

After adjusting for age, PLHIV compared to HIV negative participants were more likely to be women, never married, have low wealth index, moderate to severe food insecurity, a history of previous tuberculosis, and be on ≥ five drugs (polypharmacy) and less likely to be overweight or obese (Table 1). PLHIV were also less likely to report hypertension and diabetes mellitus (Table 1).

### The 95-95-95 targets

**Fig. 2:**
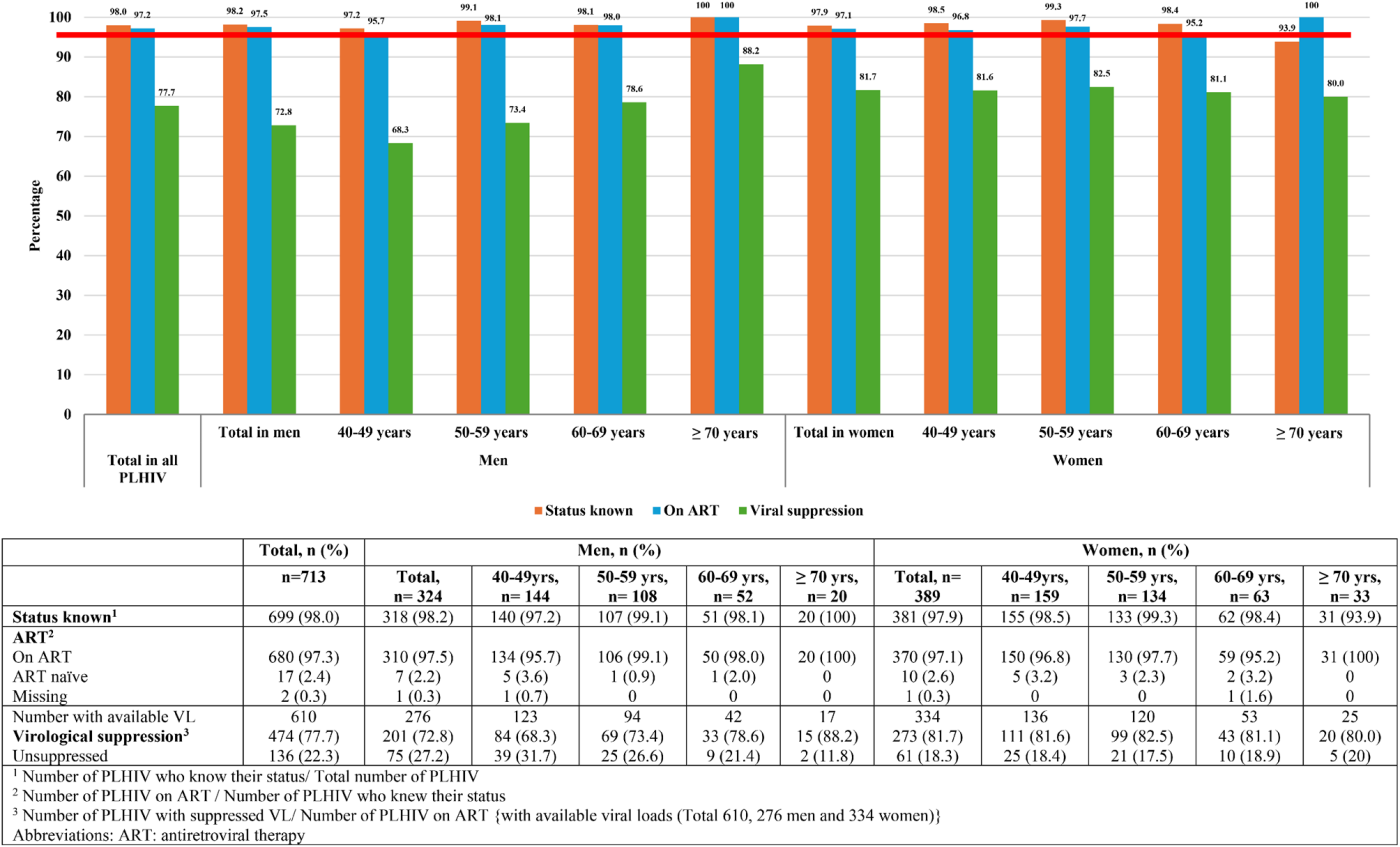
95-95-95 targets by sex and age bands

Overall, the UNAIDS 95-95-95 targets were met for study participants knowing their status (98.0% (95%CI 96.8–98.8%)) and being on treatment (97.6% (95%CI 96.2–98.4%)), but not for virological suppression (77.7% (95%CI 74.4–81.0)). These results varied by sex and age groups (Fig. 2).

Known status was highest in men ≥ 70 years at 100%. In women, with an exception of women ≥ 70 years, where only 93.9% (95%CI 85.8–100)) knew their status, all other age categories met the 95% target.

All age categories in both sexes met the treatment target with 100% of the ≥ 70 years old men and women who knew their status being on treatment.

No age or sex category met the 95% virological suppression target. Older groups had better virological suppression with the highest proportion of virological suppression (88.2% (95%CI 72.2–100) seen in men age ≥ 70 years, although women generally had a better virological suppression across all age groups of ≥ 80%. The poorest virological suppression was seen in men aged 40–49 years at 68.3% (Fig. 2).

Women (278 (57.8%), OR 1.6 (95%CI: 1.1–2.3), *p* = 0.009), those from the rural area (236 (49.1%), OR 1.5 (95%CI: 1.0-2.1); *p* = 0.044), ≥ 3 years since diagnosis of HIV (299 (62.2), OR 3.1 (95%CI: 1.6–5.8); *p* = 0.001), and those on a tenofovir, lamivudine and dolutegravir (TLD) drug combination (299 (62.2%), OR 1.6 (95%CI: 1.1–2.3); *p* = 0.012) were more likely to be virologically suppressed after adjusting for age. Duration of ART was not associated with viral suppression; however, those on second line treatment regimens were less likely to be virologically suppressed (OR 0.1 (95%CI: 0-0.3); *p* < 0.001) (Table 2).

**Table 2:** Comparison of sociodemographic and clinical characteristics between PLHIV virologically suppressed and unsuppressed

	Suppressed 481 (75.5%)	Unsuppressed 156 (24.5%)	Unadjusted OR (95% CI) of being suppressed		Adjusted for age OR (95% CI) of being suppressed		Multivariable analysis*	
	VL <50 copies/mL	VL ≥ 50 copies/mL	OR 95% CI	p value	AOR 95% CI	p value	OR 95% CI	p value
Age, median [IQR]	51 [46-59]	50 [45-58]	1.0 (0.9-1.0)	0.424				
Men, n (%)	203 (42.2)	85 (54.5)	reference		reference			
Women, n (%)	278 (57.8)	71 (45.5)	1.6 (1.1-2.4)	0.008	1.6 (1.1-2.3)	0.009	1.6 (1.0-2.6)	0.053
Living alone, n (%)	54 (11.2)	20 (12.8)	0.9 (0.5-1.5)	0.59	0.9 (0.5-1.5)	0.581		
Never married, n (%)	319 (66.3)	110 (70.5)	reference		reference			
Married/ Cohabitating, n (%)	110 (22.9)	31 (19.9)	1.2 (0.8-1.9)	0.795	1.2 (0.8-1.9)	0.872		
Divorced/Separated, n (%)	22 (4.6)	7 (4.5)	1.1 (0.5-2.6)		1.0 (0.4-2.5)			
Widowed, n (%)	30 (6.2)	8 (5.1)	1.3 (0.6-2.9)		1.2 (0.5-2.8)			
None/ Primary education, n (%)	129 (26.8)	36 (23.1)	reference		reference			
Secondary/Tertiary education, n (%)	352 (73.2)	119 (76.3)	1.2 (0.8-1.9)	0.375	1.2 (0.7-1.9)	0.522		
Urban, n (%)	245 (50.9)	94 (60.3)	reference		reference			
Rural, n (%)	236 (49.1)	62 (39.7)	1.4 (1.0-2.1)	0.043	1.5 (1.0-2.1)	0.044		
Employed, n (%)	387 (80.5)	126 (80.8)	reference		reference			
Unemployed, n (%)	94 (19.5)	30 (19.2)	1.0 (0.6-1.6)	0.932	1.1 (0.7-1.7)	0.815		
Low wealth index, n (%)	221 (45.9)	83 (53.2)	reference		reference			
Middle wealth index, n (%)	203 (42.2)	61 (39.1)	1.2 (0.9-1.8)	0.181	1.2 (0.9-1.8)	0.191		
High wealth index, n (%)	57 (11.9)	12 (7.7)	1.7 (0.9-3.5)		1.8 (0.9-3.5)			
No tobacco use (Past and never)	342 (71.3)	103 (66.0)	reference		reference			
Current tobacco use	137 (28.5)	53 (34.0)	0.8 (0.5-1.1)	0.198	0.8 (0.5-1.2)	0.243		
Alcohol, n (%)								
None	280 (58.2)	83 (53.2)	reference		reference			
Drinks alcohol	200 (41.5)	73 (46.8)	0.8 (0.6-1.2)	0.261	0.8 (0.6-1.2)	0.339		
Food insecurity, n (%)								
None to mild (score 0-2)	202 (42.0)	56 (35.9)	reference		reference			
Moderate to severe (score 3-5)	279 (58.0)	100 (64.1)	0.8 (0.5-1.1)	0.178	0.8 (0.5-1.1)	0.18		
Comorbidity, n (%)								
Hypertension	206 (42.8)	58 (37.2)	1.2 (0.9-1.8)	0.266	1.2 (0.8-1.8)	0.33		
Diabetes	38 (7.9)	10 (6.4)	1.2 (0.6-2.5)	0.566	1.2 (0.6- 2.4)	0.609		
Depression and anxiety	57 (11.9)	17 (10.9)	1.0 (0.6-2.0)	0.747	1.1 (0.6-2.0)	0.68		
Tuberculosis	100 (20.8)	38 (24.4)	0.8 (0.5-1.2)	0.334	0.8 (0.5-1.2)	0.337		
Polypharmacy, n (%)	79 (16.4)	21 (13.5)	1.3 (0.8-2.1)	0.378	1.2 (0.7-2.1)	0.453		
BMI categories, n (%)								
Underweight: < 18.5 kg/m^2^	20 (4.2)	10 (6.4)	0.9 (0.4-1.9)		0.9 (0.4-1.9)			
Normal: 18.5-24.9 kg/m^2^	151 (31.4)	65 (41.7)	reference		reference			
Overweight: 25-29.9 kg/m^2^	102 (21.2)	30 (19.2)	1.5 (0.9-2.4)	0.1618	1.4 (0.8-2.3)	0.2136		
Obese class I: 30-34.9 kg/m^2^	94 (19.5)	22 (14.1)	1.8 (1.1-3.1)		1.8 (1.1-3.1)			
Obesity class II: 35-39.9 kg/m^2^	49 (10.2)	14 (9.0)	1.5 (0.8-2.9)		1.5 (0.8-2.9)			
Morbidly obese (class III): ≥ 40kg/m^2^	53 (11.0)	13 (8.3)	1.8 (0.9-3.4)		1.8 (0.9-3.4)			
≥ 3 years since diagnosis	299 (62.2)	86 (55.1)	3.0 (1.6-5.7)	0.001	3.1 (1.6-5.8)	0.001	3.0 (1.6-6.0)	0.001
Duration on ART (years) [median (IQR)]	10 [7-14]	10 [5-14]	1.0 (0.9-1.0)	0.347	1.0 (0.9-1.0)	0.411		
ART combination, n (%)								
TEE	38 (7.9)	12 (7.7)	1.0 (0.5-2.0)	0.941	1.0 (0.5-2.0)	0.948		
TLD	299 (62.2)	78 (50.0)	1.6 (1.1-2.3)	0.013	1.6 (1.1-2.3)	0.012	2.0 (1.2-3.3)	0.006
ALD	11 (2.3)	3 (1.9)	0.9 (0.3-2.8)	0.843	0.9 (0.3-2.8)	0.807	0 )	
DTG based regimen	54 (11.2)	9 (5.8)	2.1 (1.0-4.2)	0.052	2.0 (0.9-4.1)	0.074	2.6 (1.1-5.7)	0.023
Second line	2 (0.4)	10 (6.4)	0.1 (0-0.3)	<0.001	0.1 (0-0.3)	<0.001	0.03 (0.004-0.3)	0.001

Abbreviations: AOR: age adjusted odds ratios, ART: antiretroviral therapy, BMI: body mass index, IQR: interquartile range, kg/m^2^: kilograms per metre squared, OR: Odds ratio,

ART combinations: TEE: tenofovir+ emtricitabine+ efavirenz, TLD: tenofovir+ lamivudine+ dolutegravir or tenofovir+ lamivudine with missing 3^rd^ drug, ALD: abacavir+ lamivudine+ dolutegravir, DTG based: on dolutegravir with 2^nd^ and 3^rd^ drug unknown, Second line: on atazanavir or lopinavir or darunavir

*Multivariable analysis: Variables with a p of 0.1 on univariate analysis included into the full model. The least significant variable eliminated from the model until only significant variables (p 0.05) were left

Full model: sex, urban/urban, years since diagnosis, TLD, DTG based regimen, second line regimen

Missing (n): level of education=1, tobacco use=1, BMI=14

### Age-associated characteristics in people with HIV

Younger adults (40–49 years) LHIV (*n* = 303) were more likely to be single, have attained a secondary or tertiary education, smoke and consume alcohol. Comparatively, older adults (≥ 50 years) LHIV (*n* = 410) were more likely to be widowed, have hypertension, diabetes, and be on ≥ five medicines (polypharmacy) (Table 3).

**Table 3:** Comparison of the sociodemographic and clinical characteristics between younger and older PLHIV

	40-49 years, n= 303	≥ 50 years, n= 410	p value
Age, median [IQR]	45 [42-47]	58 [53-64]	
Never married, n (%)	234 (77.2)	236 (57.6)	
Married/ Cohabitating, n (%)	59 (19.5)	105 (25.6)	
Divorced/Separated, n (%)	6 (2.0)	25 (6.1)	<0.001
Widowed, n (%)	4 (1.3)	44 (10.7)	
Living alone, n (%)	60 (11.0)	22 (13.1)	0.459
None/ Primary education, n (%)	32 (10.6)	159 (38.8)	
Secondary/Tertiary education, n (%)	270 (89.1)	251 (61.2)	<0.001
Missing	1 (0.3)		
Urban n (%)	144 (47.5)	206 (50.2)	
Rural n (%)	159 (52.5)	204 (49.8)	0.473
Low wealth index, n (%)	156 (51.5)	185 (45.1)	
Middle wealth index, n (%)	115 (38.0)	172 (42.0)	0.226
High wealth index, n (%)	32 (10.6)	53 (12.9)	
Not smoking (Past and never)	199 (65.7)	304 (74.2)	
Current smoking	104 (34.3)	105 (25.6)	0.012
Missing		1 (0.2)	
None (Alcohol), n (%)	151 (49.8)	269 (65.6)	
Drinks alcohol, n (%)	151 (49.8)	141 (34.4)	<0.001
Missing	1 (0.3)		
None to mild food insecurity (score 0-2)	128 (42.2)	182 (44.4)	
Moderate to severe food insecurity (score 3-5)	175 (57.8)	228 (55.6)	0.568
On ART	284 (93.7)	396 (96.6)	
ART naïve	10 (3.3)	7 (1.7)	0.167
Missing	9 (3.0)	7 (1.7)	
Virologically suppressed	200 (73.0)	281 (77.4)	0.2
Comorbidity, n (%)			
Hypertension	91 (30.0)	209 (51.0)	< 0.001
Diabetes	15 (5.0)	41 (10.0)	0.016
Depression and anxiety	39 (12.9)	40 (9.8)	0.191
Tuberculosis	55 (18.2)	90 (22.0)	0.215
Polypharmacy	28 (9.2)	83 (20.2)	< 0.001
Normal (MUAC ≥ 24 cm)	175 (57.8)	239 (58.3)	
Malnutrition (MUAC <24 cm)	121 (39.9)	157 (38.3)	0.744
Missing	7 (2.3)	14 (3.4)	
MUAC (median [IQR])	25 [22-30]	25.1 [21.1-28.9]	0.177
BMI (median [IQR])	26.2 [21.9-34.8]	27.8 [23.0-33.3]	0.797
BMI categories, n (%)			
Underweight: < 18.5 kg/m^2^	25 (4.6)	8 (4.8)	
Normal: 18.5-24.9 kg/m^2^	190 (34.8)	51 (30.4)	
Overweight: 25-29.9 kg/m^2^	108 (19.8)	40 (23.8)	0.288
Obese class I: 30-34.9 kg/m^2^	98 (18.0)	33 (19.6)	
Obesity class II: 35-39.9 kg/m^2^	49 (9.0)	22 (13.1)	
Morbidly obese (class III): ≥ 40kg/m^2^	62 (11.4)	12 (7.1)	
Missing	13 (2.4)	2 (1.2)	

Abbreviations: ART: antiretroviral therapy, BMI: body mass index, cm: centimetre, IQR: interquartile range, MUAC: mid upper arm circumference, kg/m2: kilograms per metre squared

## Discussion

KwaZulu-Natal has the highest prevalence of HIV in SA; however, to date studies have mainly included persons aged 15–49 years with one study which compared viral suppression between urban and rural KZN [33]. In this study, we report a prevalence of HIV in adults age ≥ 40 years of 37% (similar in men and women, and urban and rural KZN), and a prevalence of 28% in persons aged ≥ 50 years. When applying the study prevalence to the population in KZN, the projected provincial age-and sex-standardised prevalence for adults aged ≥ 40 years was 47.4%, a little higher in women compared to men (49% vs. 45%). Given that these projections assume homogeneity in prevalence across the province, they should not be over-interpreted.

Several reasons for higher prevalence in women have been postulated and include possible increased susceptibility to HIV infection, lower declines in female incidence [3], or an increased willingness to participate in research studies. In 2024 the Human Science Research Council reported a higher prevalence of HIV amongst younger women (25–49 years) in KZN compared to their male counterparts (38.4% vs. 21.5%), however, this was substantially lower than our study prevalence of 75.4% in women aged 40–49 years. This could be due to participation bias, overrepresentation of clinic-engaged individuals and/or demographic clustering in our study populations. Whilst UNAIDS 95-95-95 targets were met for known status and being on treatment (98.0% and 97.6%, respectively), they were not for virological suppression (77.7%). Better virological suppression was seen in older participants, women and those with longer duration since diagnosis with HIV.

The prevalence of HIV in persons ≥ 50 years of 28% is higher than previously reported in Swaziland (13%), Ethiopia (6.2%) and Kenya (5%) [5, 9, 34] and almost three times higher than previously reported in northern KZN in 2007 (9.4%) and 2008 (9.5%) [6]. While a higher prevalence (16.5%) was reported in a subsequent study in a rural setting in Mpumalanga province in 2010 [7], this is still substantially lower than in our study. This temporal increase in prevalence may be explained by improved access to ART and health care resulting in an increased survival in PLHIV [3], new HIV infections, and/or improved detection in older persons. Over 97% of older PLHIV in this study were on ART with only five diagnosed during the study.

The higher percentage of virological suppression in older people, concurs with previous American and European studies, which attributed this to better medication adherence [35–38] but could also be due to survival bias, differential healthcare engagement, and/or diagnostic intensity. Achievement of the 95-95-95 targets in this study (98-98-78) was better than previously reported for the 90-90-90 targets in SA in 2017 which were (85-71-88) in those aged 15 to 64 years [11, 39] and the 95-95-95 targets in March 2024 SANAC report which were (95-79-74) in the total population [12]. With the exception of the viral suppression, the targets in this study were slightly higher than that reported in Zimbabwe (93-96-90) in the same age groups [13]. In addition, the proportion who knew their status (98%) and were on ART (97%) exceeded the overall figures for known status (86% and 95%) and being on ART (89% and 79%) in 2024 UNAIDS worldwide and SANAC reports. However, the proportion of those who achieved virological suppression at 77.7% in this study, was lower than the 93% in the World Report and higher than the 74% in the SANAC report [3, 12]. The lower attainment of virological suppression, despite reported ART use in our study, may be explained by poor adherence and participation bias, as other factors described in literature like marital and education status were not associated with suppression status in this study [40].

A higher proportion of women achieving virological suppression is similar to previous reports in SA and Tanzania [41, 42]. Women achieving better virological suppression than men has been postulated to be due to biological differences with men having higher levels of viraemia than women [43] and behavioural factors with men having 1.73 times odds of non-adherence than women [44].

Previously in KZN, no differences were reported in treatment outcomes between urban and rural areas, although PLHIV in a peri-urban area achieved earlier virological suppression [33]. In contrast, in this study PLHIV in the rural area were more likely to be virologically suppressed. While there were no differences in participant characteristics between the urban and rural sites, the rural recruitment was in proximity to the Centre for the AIDS Programme of Research in South Africa (CAPRISA), meaning the population had access to specialist HIV care. Robust patient tracking systems and comprehensive care programmes may have enhanced treatment adherence and viral suppression outcomes [45].

With growing economic development, the prevalence of chronic NCDs is increasing across Africa, including SA; the most common being hypertension reported in 46% in women and 44% in men in the South Africa Demographic and Health Survey (SADHS) [46]. Despite variance between countries, type 2 diabetes mellitus is also common, with the highest prevalence across Africa seen in urban SA [47]. The high prevalence of hypertension and diabetes in the study population reflects that expected of an older general population. Although HIV infection and ART is thought to increase cardiovascular risk, this was not evident in our study. The prevalence of hypertension in PLHIV in our study (42.1%) was close to the 38.2% reported by Roomaney et al. [8] and lower than the 50.1% reported by Okyere et al. A similar prevalence has also been reported in other African countries [34, 48–52]. Similarly, the higher prevalence of diabetes in participants who were HIV negative (18.7%) is likely a reflection of their older age.

Although polypharmacy was higher in PLHIV compared to the HIV negative group (15.6% vs. 9.6%) in this study, this was lower than the pooled prevalence of 33% (25–42%) reported in a systematic review and meta-analysis on prevalence and global trends of polypharmacy among PLHIV [53]. The higher prevalence of polypharmacy in PLHIV can be explained by being on ART, which adds three drugs to others that participants may be on. An addition of two drugs for any NCD would then classify them as having polypharmacy. The higher burden of NCDs may also explain the higher likelihood of polypharmacy in the older group of PLHIV [54].

Integrated clinics for HIV and hypertension and diabetes, have been reported to reduce HIV-related stigma [55–58]. Including care of the multiple chronic conditions in HIV clinics might be a much-needed adjustment in management of older PLHIV in SA.

The country has seen marked reductions in new HIV infections and improved ART uptake [12] which together with improved primary health care have contributed to improved life expectancy and therefore PLHIV are now living longer with increased risk of NCDs and polypharmacy.

### Strengths and limitations

The strength of this study lies in it being community-based across different age bands, including those aged ≥ 65 years. Although self-reported history of NCDs is often used, this may introduce recall bias. We mitigated against this by physical assessment and POC blood tests (blood pressure and glucometer readings) and a review of medication. There may also be a recall bias for the length of time since diagnosis of HIV.

The large sample size and inclusion of a large number of PLHIV in this study as well as inclusion of both urban and rural populations allows for generalization to the rest of KZN province, but not the whole country, given the differences in HIV prevalence between provinces. However, we assumed the study HIV prevalence estimates were generalizable to the province, which may not have been the case. For example, our study may have been subject to participation bias, as PLHIV may be more concerned about their health and therefore more likely to enroll in a study, leading to overestimates of HIV prevalence, or, conversely, overrepresentation of those engaged in healthcare with known HIV status, and an under-representation of those with undiagnosed HIV, which would lead to underestimation of the true prevalence. Notably, only a small percentage of participants were newly diagnosed with HIV during the study. It is also possible that HIV prevalence varies across the province due to other factors, such as access to health care, socioeconomic and behavioral factors.

The proportion that were virologically suppressed was slightly lower in those with VL taken during the study compared to those with VL retrieved from NHLS (72.4% and 80.4%, respectively). The median (IQR) period from the last VL to the study clinic visit was 3 months (0–6 months). This could mean that those who have regular checkups with results from NHLS were more compliant. However, it is possible that the participants who had their VLs retrieved from NHLS might have changed their status in the interim between testing at NHLS and the study clinic.

## Conclusion

With the expansion of the ART rollout, PLHIV are living longer. This is particularly relevant in KZN, which has the highest prevalence of HIV in SA. In this study we report a HIV prevalence of 27.9% among adults aged ≥ 50 years, and overall UNAIDS 95-95-95 targets of 98-98-78. Although targets were not met overall, higher levels of viral suppression were noted in women in all age groups and men ≥ 70 years. Greater efforts are needed to achieve virological suppression in South Africa. Furthermore, the high prevalence of chronic non-communicable diseases in older PLHIV highlights the importance of integrated care.

## Supplementary Information

Below is the link to the electronic supplementary material.

Supplementary Material 1

Supplementary Material 2

## Data Availability

The data set used for this paper is under ethical restriction imposed by the Biomedical Research Ethics Committee (BREC) of the University of KwaZulu-Natal, which stipulates that a South African co-author is included in all publications resulting from data collected on South African human subjects. Third party researchers wishing to access individual data records, will need to submit an ethically approved protocol to the Study Principal Investigator (Professor Celia Gregson) and in-country Principal Investigator (Professor Bilkish Cassim) for subsequent approval by BREC [https://research.ukzn.ac.za/] (https:/research.ukzn.ac.za/%20research-office) ethics-overview/biomedical-research-ethics/Contact information: [BREC@ukzn.ac.za] (mailto: BREC@ukzn.ac.za) , +27 312602486Senior administrative officer: Anusha Marimuthu, [marimuthu@ukzn.ac.za] (mailto: marimuthu@ukzn.ac.za), +27 312604769Assistant administrative officer: Nompumelelo Zulu, [Zulu4@ukzn.ac.za] (mailto: Zulu4@ukzn.ac.za) , +27312601074Data will be stored on a secure password protected University of Bristol network filestore space for ten years beyond the completion of the study, ending on 30th September 2036. Data for sharing will be anonymised and contain no information that could directly identify any of the participants. Data users will need to acknowledge data sources and ensure regulatory requirements of the South African BREC are met.

## Abbreviations

AIDS: Acquired immunodeficiency syndrome
ALD: Abacavir+ lamivudine+ dolutegravir
ART: Antiretroviral therapy
BMI: Body mass index
CAPRISA: Center for The AIDS Programme of Research In South Africa
cm: Centimetres
Fractures-E^3^: Fractures in Sub-Saharan Africa: epidemiology, economic impact and ethnography
HIC: High income countries
HIV: Human immunodeficiency virus
AIC: Akaike Information Criterion
IQR: Interquartile range
kg: Kilograms
kg/m^2^: Kilograms per metre squared
LE: Life expectancy
LHIV: Living with HIV
LMIC: Lower middle income
MUAC: Mid-upper arm circumference
m/s: Metres per seconds
NCD: Non-communicable diseases
PLHIV: People living with HIV
REDCap®: Research Electronic Data Capture
s: Seconds
SADHS: South Africa Demographic and Health survey
SANAC: Department of Health South African National AIDS council
SSQ: Shona symptom questionnaire
SD: Standard deviation
TEE: Tenofovir+ emtricitabine+ efavirenz
TLD: Tenofovir+ lamivudine+ dolutegravir
UNAIDS: Joint United Nations Programme on HIV/AIDS
US: United States on America
WS: Walking speed
