# Supplementary material for: UNAIDS 95-95-95 targets in older people living with HIV in urban and rural KwaZulu-Natal, South Africa
Source: BMC Infect Dis. 2026 Apr 7;26:972. doi: 10.1186/s12879-026-13273-y (PMC13188679; doi:10.1186/s12879-026-13273-y)
Supplement: Supplementary file 1 — Supplementary Material 1 [file 12879_2026_13273_MOESM1_ESM.docx]

**Supplementary Figure**

**Supplementary Figure 1**

| 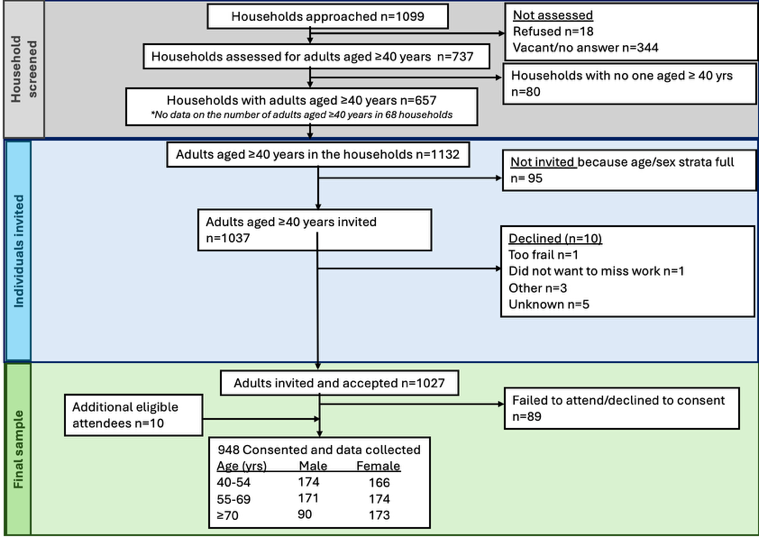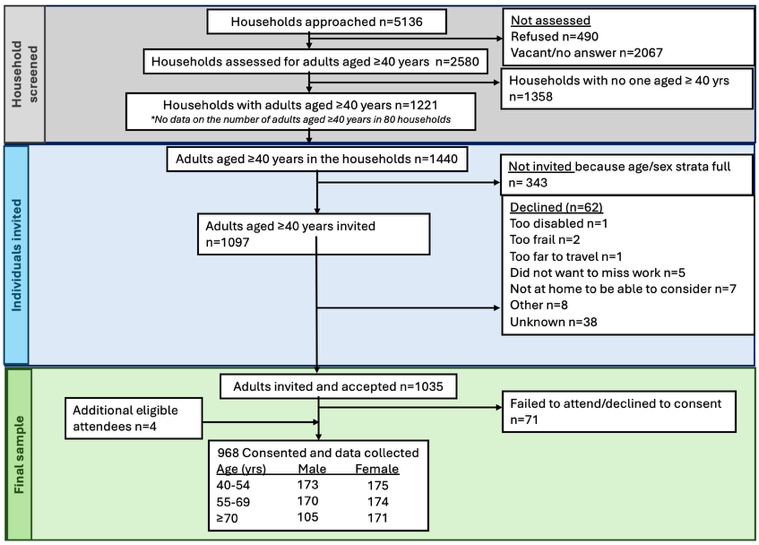  B  A |
| --- |

**Supplementary Figure 1: Flow diagrams of urban (A) and rural (B) sites.**
