# Supplementary material for: UNAIDS 95-95-95 targets in older people living with HIV in urban and rural KwaZulu-Natal, South Africa
Source: BMC Infect Dis. 2026 Apr 7;26:972. doi: 10.1186/s12879-026-13273-y (PMC13188679; doi:10.1186/s12879-026-13273-y)
Supplement: Supplementary file 2 — Supplementary Material 2 [file 12879_2026_13273_MOESM2_ESM.docx]

| **MODULE 1: Demographic Data** | | | |
| --- | --- | --- | --- |
| **001** | *STUDYID* | Study ID | □□□□ |
| **002** | *MYB* | Month and year of birth | MM/19YY |
| **003** | *GEN* | Gender of patient | 1=Male 2=Female |
| **004** | *DOC* | Date of interview | □□/□□/20□□ |
| **005** | *OID* | Interviewer ID | □□□□ |

| **MODULE 2: Socio-economic and household characteristics** | | | | | | |
| --- | --- | --- | --- | --- | --- | --- |
| **006** | *NOHH* | How many people live in your household? Include all people who regularly live and share meals in the house. Regularly meaning someone who has lived at the house for at least 4 weeks (one month). Include all adults, children and babies and yourself | | | | □□ |
| **007_n & 008_n** | *ASHH* | Please answer the following for each person living in your household, starting with yourself  1 Age □□ years Sex (1=Male 2=Female) □  2 Age □□ years Sex (1=Male 2=Female) □  3 Age □□ years Sex (1=Male 2=Female) □  4 Age □□ years Sex (1=Male 2=Female) □  5 Age □□ years Sex (1=Male 2=Female) □  6 Age □□ years Sex (1=Male 2=Female) □  7 Age □□ years Sex (1=Male 2=Female) □  8 Age □□ years Sex (1=Male 2=Female) □  9 Age □□ years Sex (1=Male 2=Female) □  10 Age □□ years Sex (1=Male 2=Female) □ | | | |  |
| **009** | *PROV* | Which province do you live in? *(Zimbabwe only)*  1= Bulawayo 2= Harare 3= Manicaland  4= Mashonaland Central 5= Mashonaland East 6= Mashonaland West  7= Masvingo 8= Matabeleland North 9= Matabeleland South  10= Midlands 11= Don’t know  Which province did you live in? *(South Africa only)*  1= Eastern Cape 2= Free State 3= Gauteng  4= KwaZulu-Natal 5= Limpopo 6= Mpumalanga  7= Northern Cape 8= North West 9= Western Cape  10 = Don’t know  Which region did you live in? *(The Gambia only)*  1= Lower River Region (LRR) 2= Central River Region (CRR)  3= North Bank Region (NBR) 4= Upper River Region (URR)  5= West Coast Region (WCR) 6= Banjul City Council (BCC)  7 = Kanifing Municipal Council (KMC)  8 = Don’t know | | | |  |
| **010** | *RUR* | Do you live in: (tick one)  A Village □ A Town □ A City □  Other □ (please specifiy) ____________ | | | |  |
| **011** | *EDM* | What is your highest level of completed education?  1 = Primary 2 = Secondary 3 = Training college  4 = University 5 = None 6 = Unknown | | | | □ |
| **012** | *OWN* | Does your household own the dwelling you live in?  1 = Own dwelling, 2 = Rent main dwelling, 3 = Rent part of dwelling/lodger, 4 = Use dwelling without paying rent | | | | □ |
| **013** | *WAL* | What is the main material of the exterior walls of your dwelling ?  1. Cement 2. Wood 3. Mud 4. Other | | | | □ |
| **014** | *ROF* | What is the roof of your dwelling made of?  1. Tile 2. Asbestos 3. Corrugated iron 4. Wood 5. Thatch 6. Other | | | |  |
| **015** | *SAL* | What would you say is the usual household income per month? This includes grants, formal and informal income.  *Zimbabwe only*:  1 = No income 2 = Less than USD 50  4 = USD 51-100 5 = USD 101-200 6 = USD 201-500  7 = USD 501-900 8 = More than USD 900 9 = Don’t know/don’t want to say  *South Africa only:*  1 = No Income 2 = Less than SAR 500 3 = SAR 500-1000  4 = SAR 1001-2000 5 = SAR 2001-3000 6 = SAR 3001-8000  7 = SAR 8001-15000 8 = More than SAR 15000 9 = Don’t know/don’t want to say  *The Gambia only:*  1 = No Income 2 = Less than GMD 2,000 3 = GMD 2,000-4,000  4 = GMD 4,001-8,000 5 = GMD 8,001- 16,000 6 = GMD 16,000-35,000  7 = GMD 35,001-60,000 8 = More than GMD 60,000  9 = Don’t know/don’t want to say | | | | □ |
| **016** | *ASS* | Does your household have any of the following? (tick all that apply)  Electricity □ Bicycle □ Television □ Working car/truck □ Tap in house □  Private water/borehole (running water) □ Flush toilet □ Pit latrine □  Solar energy □ Power generator □ Tiled floors □ Computer Internet access (WiFi) □ | | | | |
| **017** | *BIC* | How many biological children have you had (this includes those who have passed away)?  *Enter zero if none and skip next question* | | | | □□ |
| **018** | *BIS* | How many of these children are still alive? | | | | □□ |
| **019** | *BOR* | Were you born in Zimbabwe? *(Zimbabwe only)*  Were you born in South Africa? *(South Africa only)*  Were you born in The Gambia? *(The Gambia only)* | | | | *Yes □ No □*  *If yes go to question 020,*  *if no go to question 021* |
| **020** | *BORP* | Which province were you born in? *(Zimbabwe only)*  1= Bulawayo 2= Harare 3= Manicaland  4= Mashonaland Central 5= Mashonaland East 6= Mashonaland West  7= Masvingo 8= Matabeleland North 9= Matabeleland South  10= Midlands 11 = Don’t know  Which province were you born in? *(South Africa only)*  1= Eastern Cape 2= Free State 3= Gauteng  4= KwaZulu-Natal 5= Limpopo 6= Mpumalanga  7= Northern Cape 8= North West 9= Western Cape  10 = Don’t know  Which region were you born in? *(The Gambia only)*  1= Lower River Region (LRR) 2= Central River Region (CRR)  3= North Bank Region (NBR) 4= Upper River Region (URR)  5= West Coast Region (WCR) 6= Banjul City Council (BCC)  7 = Kanifing Municipal Council (KMC)  8 = Don’t know | | | | □□  *Now skip to question 023* |
| **021** | *BORC* | Which country were you born in? | | _____________ | | |
| **022** | *LDUR* | How long have you lived in Zimbabwe? *(For Zimbabwe only)*  How long have you lived in South Africa? *(For South Africa only)*  How long have you lived in The Gambia? *(For Gambia only)* | | □□ months □□ years | | |
| **023** | *ETH* | What is your ethnictiy? *(Zimbabwe only)*  1 = Black African 2 = White 3 = South Asian  4 East Asian5 = Mixed Race 6 = Other  What is your ethnictiy? *(South Africa only)*  1 = Black African 2 = White European 3 = Indian (Asian)  4 = Mixed Race 5 = Other 6 = Coloured  What is your ethnictiy? *(The Gambia only)*  1=Mandinka 2=Wollof 3=Fula 4=Jola  5=Serere 6=Serahule 7=Manjago 8=Other state………………… | | | | □ |
| **024** | *REL* | What is your religion?  1= Christian (Pentecostal) 2= Christian (Protestant)  3 = Christian (Apostolic) 4 = Christian (Roman Catholic)  5 = Muslim 6 = African Traditional/Folk  7 = Other | | □  If other, (Specify)_____________ | | |
| **025** | *MAR* | What is your marital status?  1 = Never Married 2 = Currently Married 3 = Separated  4 = Divorced 5 = Widowed 6 = Cohabitating  7 = Decline to answer | | | | □ |
| **026** | *EMP* | Do you have a job (either employed or self-employed)? | | | Formally employed □  Self-employed □  Informal job □  No job □ | |
| **027** | *JOB* | What is your job? | | | _________________ | |
| **028** | *INF* | Is your job formal? | Yes □ No □ If no, skip to next module | | | |
| **029** | *DYW* | How many days do you work per week for which you get paid? | | | | □□ days |

| **MODULE 3: Falls and Broken Bones** | | | | |
| --- | --- | --- | --- | --- |
| **030** | *FAL* | In the past year, how many times have you fallen over? | □□ | |
| **031** | *MHP* | Did your mother ever break her hip? | Yes □ No □ Don’t know □ | |
| **032** | *DHP* | Did your father ever break his hip? | Yes □ No □ Don’t know □ | |
| **033** | *CHB* | As a child, did you ever break a bone? **This includes all fractures, cracks, chips and breaks.** | Yes □ No □ Don’t know □ | |
| **034** | *ADB* | As an adult, have you ever broken a bone? **This includes all fractures, cracks, chips and breaks.** If no, proceed to 067 | Yes □ No □  Don’t know □ | |
| **035** | *BRK* | **Thinking about the broken bones you have had as an ADULT, this includes all fractures, cracks and breaks:** | | |
| **036-048** | *BBN* | **Have you ever broken a bone in the:** |  | |
|  |  | (036) Left arm/shoulder | Yes □ No □ | |
|  |  | (037) Right arm/shoulder | Yes □ No □ | |
|  |  | (038) Left wrist | Yes □ No □ | |
|  |  | (039) Right wrist | Yes □ No □ | |
|  |  | (040) Left hip | Yes □ No □ | |
|  |  | (041) Right hip | Yes □ No □ | |
|  |  | (042) Left leg | Yes □ No □ | |
|  |  | (043) Right leg | Yes □ No □ | |
|  |  | (044) Left ankle | Yes □ No □ | |
|  |  | (045) Right ankle | Yes □ No □ | |
|  |  | (046) Pelvis | Yes □ No □ | |
|  |  | (047) Spine | Yes □ No □ | |
|  |  | (048) Other (specifiy) ______________ | Yes □ No □ | |
| Multiple repeating items (n) to be completed for each broken bone: | | | | |
| **X_01_n**  (where X is 036 – 048 if answered ‘yes’) | *MEC* | For each broken bone reported: **How did you get injured?**  1 = Slip, Trip or Fall from a standing height or less  2 = Fall from height (e.g. ladder, stairs)  3 = Road traffic accident 4 = Pushed, kicked or injured by someone else  5= I can’t remember 6 = Other (please specify) _____________ | | □ |
| **X_02_n**  (where X is 036 – 048 if answered ‘yes’) | *MAN* | For each broken bone reported: **How was the broken bone managed (tick all that apply)?**  X-Ray □ Operation□ Plaster Cast/Splint □ Hospital admission □  Traditional bonesetter □ Bandage only □ Nothing □ | | |

| **MODULE 4: General health and co-morbidities** | | | | | |
| --- | --- | --- | --- | --- | --- |
| **049** | *GHQ* | In general, how would you rate your health today*?*  1=Very good 2=Good 3=Moderate 4=Bad 5=Very bad | | | □ |
| **050** | *GHC* | How has your overall health changed over the **last 12 months**?  1=Much better 2=Better 3=The same 4=Worse 5=Much worse | | | □ |
| **051** | *ADM* | How many times have you been admitted to hospital in the **last 12 months?** | | | □ □ |
| **052** | *FOR* | Have you been more forgetful in the **last 12 months** to the extent that it has significantly affected your daily life? | | | Yes □  No □ |
| **053** | *WTL* | Have you, or those close to you, noticed that you have lost weight or become thinner over the **last 12 months**? If no, proceed to *056* | | Yes □ No □ | |
| **054-055** | *KID* | How much weight did you loose? Please estimate how much in kg? | | □ □.□ kg  unable to say □ | |
| **056** | *HTL* | Have you lost height as you have grown older? If so, how much have you lost?  1= No height loss 2=Less than 2cms  3=2-4 cms 4=4-6cms  5=6cm or more (show on a ruler height lost) | | □ | |
| **057** | *ARTH* | Have you ever been diagnosed with arthritis or a joint problem? | | Yes □  No □  Don’t know □ | |
| **058** | *OSTEO* | If yes, has a doctor ever told you that you have osteoarthritis? | | Yes □  No □  Don’t know □ | |
| **059** | *JOSI* | If yes, which joint(s)/site(s)? (tick all that apply)  Left hip □ Right hip □ Left knee □ Right knee □ Hand □  Spine □ Other □ | | | |
| **060** | *HPR* | Have you undergone hip replacement surgery for arthritis? | | Yes □  No □  Don’t know □ | |
| **061** | *HPRS* | If YES, which hip? | | Left □  Right □  Both □ | |
| **062** | *HPRA* | If YES, at what age did you have the first hip replacement surgery? | | □□ years | |
| **063** | *KNR* | Have you undergone knee replacement surgery for arthritis? | | Yes □  No □  Don’t know □ | |
| **064** | *KNS* | If YES, which knee? | | Left □ Right □ Both □ | |
| **065** | *KNRA* | If YES, at what age did you have the first knee replacement surgery? | | □□ years | |
| **066** | *STER* | Have you ever taken tablet steroids (e.g. Prednisolone) for 3 months or more in your adult life?  1 = Yes, currently taking 2 = Yes, taken in the past 3 = No  4 = Don’t know | | □ | |
| Have you ever been diagnosed with any of the following (check hand held medical record)?: | | | | | |
| **067** | *EPI* | | Epilepsy | Yes □ No □ Don’t know □ | |
| **068** | *KID* | | Kidney disease | Yes □ No □ Don’t know □ | |
| **069** | *TB* | | Tuberculosis (TB) | Yes □ No □ Don’t know □ | |
| **070** | *ASTH* | | Asthma, emphysema or breathing problems | Yes □ No □ Don’t know □ | |
| **071** | *DIAB* | | Diabetes | Yes □ No □ Don’t know □ | |
| **072** | *HEART* | | Heart problem | Yes □ No □ Don’t know □ | |
| **073** | *HBP* | | High blood pressure | Yes □ No □ Don’t know □ | |
| **074** | *CHO* | | High cholesterol | Yes □ No □ Don’t know □ | |
| **075** | *THY* | | Thyroid disease | Yes □ No □ Don’t know □ | |
| **076** | *CAN* | | Cancer; If no proceed to X, if yes please specify the type of cancer: __________________________________ | Yes □ No □  Don’t know □ | |
| **077** | *STK* | | Stroke or a ‘mini-stroke’ | Yes □ No □ Don’t know □ | |
| **078** | *DEM* | | Dementia | Yes □ No □ | |
| **079** | *RHE* | | Rheumatoid Arthritis | Yes □ No □ | |
| **080** | *OST* | | Osteoporosis (brittle/fragile bones) | Yes □ No □ Don’t know □ | |
| **081** | *BAL* | | Poor balance | Yes □ No □ Don’t know □ | |
| **082** | *OBI* | | Weight problems and/or obesity | Yes □ No □ Don’t know □ | |
| **083** | *BOW* | | Bowel disease | Yes □ No □ Don’t know □ | |
| **084** | *MOT* | | Do you have any other medical problems? If Yes, please specify: ____________________ | Yes □  No □  Don’t know □ | |

| **MODULE 5: HIV History** | | | | | | | | |
| --- | --- | --- | --- | --- | --- | --- | --- | --- |
| **085** | *TEN* | Have you ever been tested for HIV? | | | | | Yes □ No □ Don’t know □ | |
| **086** | *TEP* | Have you ever tested positive for HIV? | | Yes □ No □ Don’t know □  ***The Gambia:***  Yes (known positives) go to question 087  No/don’t know go to next module  ***Zimbabwe and South Africa: POC test***  Tested and confirmed positive: go to question 087  Tested and confirmed negative: go to next module | | | | |
| **087** | *DOD* | When was your HIV diagnosed? | | | | | | Date: MM/YYYY |
| **088 – 089** | *RCD* | What was your most recent CD4 count (if known)? | | | | | | □,□□□cells/mm^3^  Date of test: MM/YYYY |
| **090 – 091** | *RVL* | What was your most recent viral load (if known)? | | | | | | □,□□□,□□□ copies per ml  Date of test: MM/YYYY |
| **092** | *ART* | Are you on ART? | | | | | | Yes □ No □ |
| **093** | *DURA* | How long have you been on ART? | | | | | | □□ months □□ years |
| **094** | *DURC* | How long have you been taking your current regimen? | | | | | | □□ months □□ years |
| Please tick the current drugs the participant is taking and give the date each drug was started (MM/YYYY): | | | | | | | | |
| **095 – 096** | *ABC* | Abacavir Yes □ No □  Date commenced: □□/□□□□ | **107 – 108** | | *KAL* | Lopinavir/ritonavir (Kaletra/Aluvia)  Yes □ No □  Date commenced: □□/□□□□ | | |
| **097 – 098** | *DRV* | Tenofovir disoproxil fumerate (TDF)  Yes □ No □  Date commenced: □□/□□□□ | **109 – 110** | | *3TC* | Lamivudine or emtricitabine  Yes □ No □  Date commenced: □□/□□□□ | | |
| **099 – 100** | *DTG* | Dolutegravir Yes □ No □  Date commenced: □□/□□□□ | **111 – 112** | | *NVP* | Nevirapine Yes □ No □  Date commenced: □□/□□□□ | | |
| **101 – 102** | *EFV* | Efavirenz Yes □ No □  Date commenced: □□/□□□□ | **113 – 114** | | *ATV* | Atazanavir /ritonavir Yes □ No □  Date commenced: □□/□□□□ | | |
| **103 – 104** | *AZT* | Zidovudine Yes □ No □  Date commenced: □□/□□□□ | **115 – 116** | | *TDF* | Tenofovir alafenamide (TAF) Yes □ No □  Date commenced: □□/□□□□ | | |
| **105 - 106** | *DAR* | Darunavir/ritonavir  Yes □ No □  Date commenced: □□/□□□□ | **117 – 118** | | OTH | Other Yes □ No □  Date commenced: □□/□□□□  (if yes please specify) __________________________ | | |

| **MODULE 6: Medicines** | | | | | | | |
| --- | --- | --- | --- | --- | --- | --- | --- |
| **119** | *ADM* | Do you currently take any medicines?  If no, proceed to next module | | | | Yes □ No □ | |
| Could you show me the medicines you are taking? Write in name and details of each medicine: | | | | | | | |
| Multiple repeating items (n) to allow all medicine to be recorded: | | | | | | | |
| **MED*_*n** | ***120*** | *NAME* | Medicine name | _____________________________________________ | | | |
|  | ***121*** | *IND* | What do you take this medicine for? | Write in condition(s) taken for: __________________  ______________________________________________ | | | |
|  | ***122*** | *DOSE* | What is the dose?  ______ | | | | |
|  | ***123*** | *FREQ* | How frequently do you take it?  Less than once per day □ once a day □ twice a day  3 times a day □ 4 times a day□ more than 4 times a day □ | | | | |
|  | ***124*** | *INI* | When did you start taking this medicine? | □□  month □□□□ year | | | |
|  | ***125*** | *TOLD* | Who told you to take this medicine?  1=Doctor 2=Nurse 3=Family member 4=I decided for myself  5=Traditional healer 6=Pharmacist 7=Drug seller (shop, market) 8=Other | | | | □ |
|  | ***126*** | *WHE* | Where did you get this medicine from?  1= Hospital 2=Health clinic 3=Private pharmacy 4=Traditional healer  5= Market/shop 6=Other | | | | □ |
|  | ***127*** | *PAY* | How much did you pay for this medicine (out of pocket)? | | Zimbabwe  □□.□□USD  The Gambia  □□.□□GMD  South Africa  □□.□□SAR | | |
| **128** | PROB | Do you ever have problems taking or remembering to take your medication?  No □                Sometimes □       Often □  All the time □ | | | | | |

| **MODULE 7: Lifestyle Factors (Smoking, Alcohol)** | | | | |
| --- | --- | --- | --- | --- |
| **Tobacco Use** | | | | |
| **129** | *ESM* | Have you ever used tobacco or smoked?  If no, skip to 135 | | Yes □ No □ |
| **130** | *CUR* | Do you currently smoke?  If no, skip to 133 | | Yes □ No □ |
| **131** | *CIG* | How many cigarettes do you smoke a day? | | □ □ |
| **132** | *PIP* | Do you smoke a pipe? | | Yes □ No □ |
| **133** | *SNF* | Do you use snuff? | | Yes □ No □ |
| **134** | *CHW* | Do you chew tobacco? | | Yes □ No □ |
| **Alcohol Use** | | | | |
| **135** | *ALC* | Do you drink alcoholic beverages?  If no, skip to next module | Yes □ No □ Don’t know □ Declined to answer □ | |
| **136** | *DYA* | Over the past 30 days, on how many days did you drink one or more alcoholic beverages? | □□ days | |
| **137** | *DNU* | How many drinks did you have, on an average day when you drank alcohol | □□ | |
| **138** | *TYP* | What type of alcoholic beverage do you usually drink? | □ beer □ wine □ spirits □ home brew □ other (please specify all that apply) | |

| **MODULE 8: Food security and diet** | | | | | | |
| --- | --- | --- | --- | --- | --- | --- |
| The next 5 questions relate to household food insecurity. In the past 4 weeks: | | | | | | |
| **139** | | FDNE | Did you worry that your household would not have enough food? | | | Yes □ No □ |
| **140** | | FDSM | Did you or any household member have to eat a **smaller** meal than you felt you needed because there was not enough food? | | | Yes □ No □ |
| **141** | | FDFM | Did you or any household member have to eat **fewer** meals in a day because there was not enough food? | | | Yes □ No □ |
| **142** | | FDSH | Did you or any household member **go to sleep at night hungry** because there was not enough food? | | | Yes □ No □ |
| **143** | | FDLV | Did you or any household member **have to eat a limited variety of foods** due to a lack of resources | | | Yes □ No □ |
| **What has been your usual diet in the past 4 weeks? In those 4 weeks, how often have you eaten the following foods?**  Code for frequency of eating - write a code in each box:  0=Never, 1=less than 4 times in past month, 2=1-2 times a week, 3=3-5 times a week, 4=Almost every day | | | | | | |
| **Code** | **Food group** | | | **Examples** | Frequency code | |
| **144** | *LEGUMES* | | | Beans, peas, nuts, seeds or foods made from any of these e.g peanut butter | □ | |
| **145** | *DAIRY* | | | Milk, lacto, cheese, cream, milk powder or yogurt | □ | |
| **146** | *MEAT* | | | Pork, beef, goat, mutton, chicken, duck, other birds, liver, kidney, heart or other organ meats | □ | |
| **147** | *EGGS* | | | Eggs | □ | |
| **148** | *FISH* | | | Fresh or dried fish especially fish with small bones e.g mackerel or kapenta | □ | |
| **149** | *OIL & MARGARINE* | | | Cooking oil or margarine (not butter) | □ | |
| **150** | *VITAMINS*  *& MINERALS* | | | Vitamin tablets, drops or syrups, calcium supplements | □ | |

| **MODULE 9: Physical Activity** | | | |
| --- | --- | --- | --- |
| The following questions are about carrying loads on your head | | | |
| **151** | LOAD | We would like to know whether you carry loads on your head, which response is best for you:    I have never carried loads on my head □  I don't currently carry loads on my head, but I used to in the past □  I occasionally (less than once per week) carry loads on my head □  I regularly (at least once per week) carry loads on my head □  **I*f answering ‘never’ then skip to question 156*** | |
| **152-153** | LOADA | How old were you when you started and stopped regularly (at least once per week) carrying loads on your head?  Age start □□ years Age stop □□ years (if applicable) | |
| **154** | LOADD | If you currently carry loads on your head, in the last 7 days, how many days have you carried a load on your head? | □□ |
| **155** | LOADP | In the last 7 days what was the longest period that you carried a load on your head? | □□hours □□mins |
| Think about all the **vigorous** activities that you did in the **last 7 days**. **Vigorous** physical activities are activities that take hard physical effort and make you breathe much harder than normal. Think *only* about those physical activities that you did for at least 10 minutes at a time. | | | |
| **156** | *VIGD* | During the **last 7 days**, on how many days did you do **vigorous** physical activities like heavy lifting, digging, running, or fast bicycling?  days per week □ or No vigorous physical activity □ (if none skip next question) | |
| **157-158** | *VIGT* | How much time did you usually spend doing **vigorous** physical activities on one of those days?  □□ hours □□ mins □ Don’t know/Not sure |  |
| Think about all the **moderate** activities that you did in the **last 7 days**. **Moderate** physical activities are activities that take moderate physical effort and make you breathe somewhat harder than normal. Think only about those physical activities that you did for at least 10 minutes at a time. | | | |
| **159** | *MODD* | During the **last 7 days**, on how many days did you do moderate physical activities like carrying light loads, or bicycling at a regular pace?  days per weeks □ or No moderate physical activities □ (if none skip next question) |  |
| **160-161** | *MODT* | How much time did you usually spend doing **moderate** physical activities on one of those days?  □□ hours □□ mins □ Don’t know/Not sure |  |
| Think about the time you spent **walking** in the **last 7 days**. This includes at work and at home, walking to travel from place to place, and any other walking that you might do just for recreation, sport, exercise, or leisure. | | | |
| **162** | *WAKD* | During the **last 7 days**, on how many days did you **walk** for at least 10 minutes at a time?  days per week □ or No walking □ (if none skip next module) |  |
| **163-164** | *WAKT* | How much time did you usually spend **walking** on one of those days?  □□ hours □□ mins □ Don’t know/Not sure |  |

| **MODULE 10: Mental Health Symptom Questionnaire** | | | |
| --- | --- | --- | --- |
| During the course of the past week...  All scored 1=yes/0=no | | | |
| **165** | *DEEP* | There were times in which I was thinking deeply or thinking about many things. | Yes □ No □ |
| **166** | *CONC* | I found myself sometimes failing to concentrate. | Yes □ No □ |
| **167** | *TEMP* | I lost my temper or got annoyed over trivial matters. | Yes □ No □ |
| **168** | *DREA* | I had nightmares or bad dreams. | Yes □ No □ |
| **169** | *SEE* | I sometimes saw or heard things which others could not see or hear. | Yes □ No □  If yes, refer to PI |
| **170** | *ACHE* | My stomach was aching. | Yes □ No □ |
| **171** | *FEAR* | I was frightened by trivial things. | Yes □ No □ |
| **172** | *SLEP* | I sometimes failed to sleep or lost sleep. | Yes □ No □ |
| **173** | *CRY* | There were moments when I felt life was so tough that I cried or wanted to cry. | Yes □ No □ |
| **174** | *RUN* | I felt run down (tired). | Yes □ No □ |
| **175** | *SUI* | At times I felt like committing suicide. | Yes □ No □  If yes, refer to PI |
| **176** | *UHAP* | I was generally unhappy with things that I would be doing each day. | Yes □ No □ |
| **177** | *WORK* | My work was lagging behind. | Yes □ No □ |
| **178** | *DEC* | I felt I had problems in deciding what to do. | Yes □ No □ |
| **179** | **TOTAL SCORE**  (If a participant scores between 8-14, inform the study PI as they will need a brief counselling intervention and referral to psychiatry for further assessment). | | □ □ |

| **MODULE 11: Disability and Pain** | | | | |  |  |
| --- | --- | --- | --- | --- | --- | --- |
| **180** | *DIS* | Do you consider yourself to be disabled? | | Yes □ No □ |  |  |
| **181** | *DES* | If yes, in what way*?* | ___________________________ | |  |  |
| **182** | *MOB* | How able are you to move about?  Freely mobile without aids □  Mobile with one stick □  Mobile with two sticks □  Mobile with frame or trolley □  Mobile with a wheelchair □  No mobility □  Unknown □ | | |  |  |
| **183** | *EYEC* | Do you wear glasses or contact lenses?  Yes, all of the time □ Yes, only for certain activities □ No, none of the time □  *Refuse to answer □ Don’t know □* | | |  |  |
| **184** | *SDIF* | Do you have difficulty seeing, even if wearing glasses or contact lenses?  No - no difficulty □  Yes – some difficulty but can clearly see someone’s face across a room □  Yes - some difficulty, including some difficultly clearly seeing someone’s face across a room □  Yes – a lot of difficulty but can clearly see someone’s face across a room □  Yes – a lot of difficulty, including a lot of difficultly clearly seeing someone’s face across a room  Cannot do at all □  *Refuse to answer □*  *Don’t know □* | | |  |  |
| **185** | *COIN* | *For South Africa only:*  Do you have difficulty clearly seeing the picture on a coin?  No - no difficulty □ Yes – some difficulty □ Yes – a lot of difficulty □ Cannot do at all □ Refuse to answer □ Don’t know □ | | |  |  |
| **186** | *EEX* | *For South Africa only:*  When did you last have your eyes examined by a professional?  □ □ years (0 for <1 year, 888 never,  999 don’t know) | | |  |  |
| **187** | *HAID* | Do you use a hearing aid?  Yes, all of the time □ Yes, only for certain activities □ No, none of the time □  Refuse to answer □ Don’t know □ | | |  |  |
| **188** | *HDIF* | Do you have difficulty hearing, even if using a hearing aid?  No - no difficulty □ Yes – some difficulty □ Yes – a lot of difficulty □ Cannot do at all □ Refuse to answer □ Don’t know □ | | |  |  |
| **189** | *OTPN* | Throughout our lives, most of us have had pain from time to time (such as minor headaches, sprains, and toothaches). Have you had pain other than these everyday kinds of pain **in the last 48 hours**? | | Yes □ No □ |  |  |
| **190_n** | *PSO* | 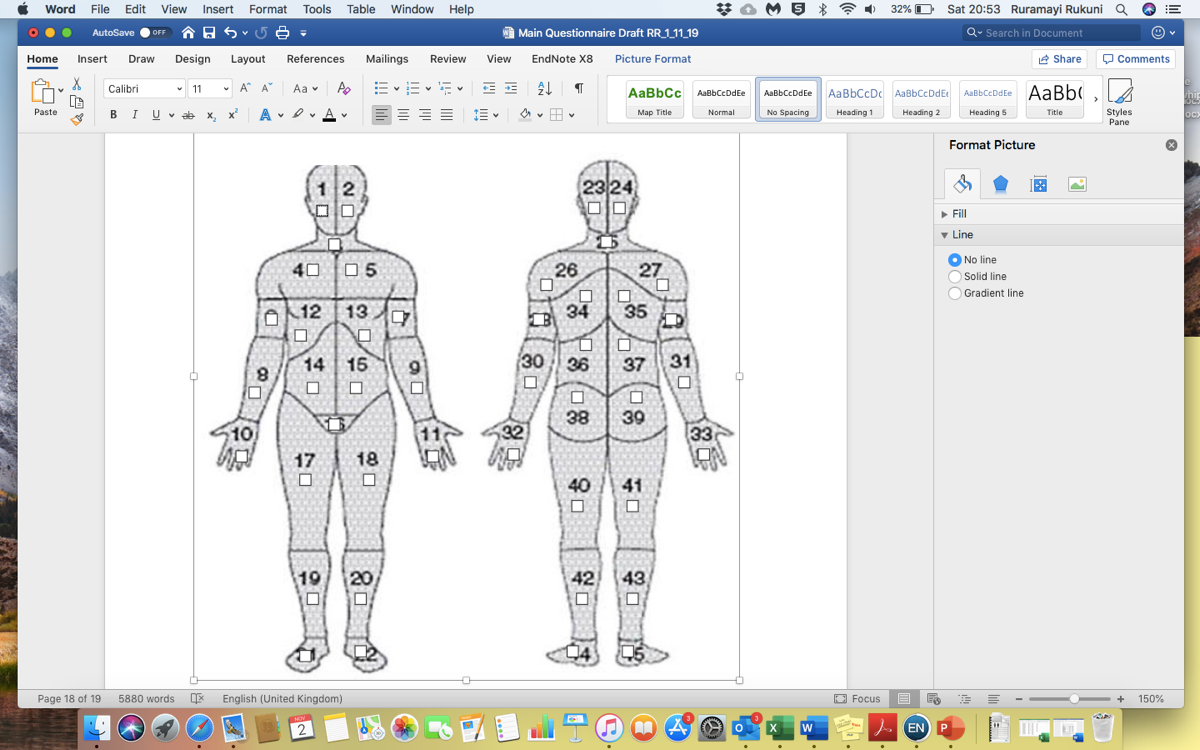Using the diagram below, please tick the box for the places where you’ve felt pain in the **last 48 hours.** | | |  |  |
|  |  | **Back pain section** | | |  |  |
| **191** | *PEXP* | How often have you experienced back pain in the last 48 hours? (tick one)  No □ Rarely □ Sometimes □ Often □ All the time  *If answer is no, go to next module, otherwise go to next question* | | |  |  |
| **192** | *PFEL* | What does your back pain feel like? (tick all that apply)  Annoying □ Dull □ Tooth ache □ Stinging □ Sharp □ | | |  |  |
| **193-196** | *PACT* | How does your back pain change with activity?  When I start doing an activity the pain builds, and builds until it’s agony and I have to stop  I generally agree □ I generally disagree □  If I’m standing up and my back pain gets worse and worse to reach a peak – then I have to sit down immediately  I generally agree □ I generally disagree □  Generally my back pain is better with activity  I generally agree □ I generally disagree □  It feels as though I am being pulled over all the time  I generally agree □ I generally disagree □ | | |  |  |
| **197-200** | *PEXT* | To what extent do the following items increase or decrease your back pain?  Walking  Increases my back pain □ Decrease my back pain □ No effect on my back pain □  Sitting on straight-backed chairs  Increases my back pain □ Decrease my back pain □ No effect on my back pain □  Sitting on soft surfaces (e.g. a cushion)  Increases my back pain □ Decrease my back pain □ No effect on my back pain □  Reclining  Increases my back pain □ Decrease my back pain □ No effect on my back pain □ | | |  |  |

| **MODULE 12: Hip and Knee Pain** | | |
| --- | --- | --- |
| The following questions concern the amount of pain you have experienced in your **knees**. For each situation please enter the amount of pain experienced in the **last 48 hours**.  Question: How much pain do you have? | | |
| **201** | *KPFLAT* | Walking on a flat surface  None □ Mild □ Moderate □ Severe □ Extreme □ |
| **202** | *KPSTAR* | Going up or down stairs  None □ Mild □ Moderate □ Severe □ Extreme □ |
| **203** | *KPBED* | At night while in bed  None □ Mild □ Moderate □ Severe □ Extreme □ |
| **204** | *KPSIT* | Sitting or lying  None □ Mild □ Moderate □ Severe □ Extreme □ |
| **205** | *KPSTND* | Standing upright  None □ Mild □ Moderate □ Severe □ Extreme □ |
| The following questions concern the amount of joint stiffness (not pain) you have experienced in the **last 48 hours** in your **knees**. Stiffness is a sensation of restriction or slowness in the ease with which you move your joints. | | |
| **206** | *KSWAK* | How **severe** is your knee stiffness **after** first **wakening** in the morning?  None □ Mild □ Moderate □ Severe □ Extreme □ |
| **207** | *KSLAT* | How **severe** is your knee stiffness after sitting, lying or resting **later in the day**?  None □ Mild □ Moderate □ Severe □ Extreme □ |
| The following questions concern your physical function. By this we mean your ability to move around and to look after yourself. For each of the following activities, please indicate the degree of difficulty you have experienced in the **last 48 hours** due to your **knees**. What degree of difficulty do you have with: | | |
| **208** | *KFSTAR* | Ascending stairs?  None □ Mild □ Moderate □ Severe □ Extreme □ No stairs climbed □ |
| **209** | *KFSIT* | Rising from sitting?  None □ Mild □ Moderate □ Severe □ Extreme □ |
| **210** | *KFFLAT* | Walking on flat?  None □ Mild □ Moderate □ Severe □ Extreme □ |
| **211** | *KFCAR* | Getting in/out of a car or bus?  None □ Mild □ Moderate □ Severe □ Extreme □ |
| **212** | *KFSOCK* | Putting on socks and/or shoes?  None □ Mild □ Moderate □ Severe □ Extreme □ |
| **213** | *KFBED* | Rising from bed?  None □ Mild □ Moderate □ Severe □ Extreme □ |
| **214** | *KFSIT* | Sitting?  None □ Mild □ Moderate □ Severe □ Extreme □ |
| **215** | *KPSLE* | In the **past week**, how much has **knee pain** affected your sleep?  Not at all □ Mildly □ Moderately □ Severely □ Extremely □ |
| The following questions concern the amount of pain you have experienced in your **hips**. For each situation please enter the amount of pain experienced in the **last 48 hours**.  Question: How much pain do you have? | | |
| **216** | *HPFLAT* | Walking on a flat surface  None □ Mild □ Moderate □ Severe □ Extreme □ |
| **217** | *HPSTAR* | Going up or down stairs  None □ Mild □ Moderate □ Severe □ Extreme □ No stairs climbed □ |
| **218** | *HPBED* | At night while in bed  None □ Mild □ Moderate □ Severe □ Extreme □ |
| **219** | *HPSIT* | Sitting or lying  None □ Mild □ Moderate □ Severe □ Extreme □ |
| **220** | *HPSTND* | Standing upright  None □ Mild □ Moderate □ Severe □ Extreme □ |
| The following questions concern the amount of joint stiffness (not pain) you have experienced in the **last 48 hours** in your **hips**. Stiffness is a sensation of restriction or slowness in the ease with which you move your joints. | | |
| **221** | *HSWAK* | How **severe** is your hip stiffness **after** first **wakening** in the morning?  None □ Mild □ Moderate □ Severe □ Extreme □ |
| **222** | *HSLAT* | How **severe** is your hip stiffness after sitting, lying or resting **later in the day**?  None □ Mild □ Moderate □ Severe □ Extreme □ |
| The following questions concern your physical function. By this we mean your ability to move around and to look after yourself. For each of the following activities, please indicate the degree of difficulty you have experienced in the **last 48 hours** due to your **hips**. What degree of difficulty do you have with: | | |
| **223** | *HFSTAR* | Ascending stairs?  None □ Mild □ Moderate □ Severe □ Extreme □ No stairs to climb □ |
| **224** | *HFSIT* | Rising from sitting?  None □ Mild □ Moderate □ Severe □ Extreme □ |
| **225** | *HFFLAT* | Walking on flat?  None □ Mild □ Moderate □ Severe □ Extreme □ |
| **226** | *HFCAR* | Getting in/out of a car or bus?  None □ Mild □ Moderate □ Severe □ Extreme □ |
| **227** | *HCSOCK* | Putting on socks and/or shoes?  None □ Mild □ Moderate □ Severe □ Extreme □ |
| **228** | *HFBED* | Rising from bed?  None □ Mild □ Moderate □ Severe □ Extreme □ |
| **229** | *HFSIT* | Sitting?  None □ Mild □ Moderate □ Severe □ Extreme □ |
| **230** | *HPSLE* | In the **past week**, how much has **hip pain** affected your sleep?  Not at all □ Mildly □ Moderately □ Severely □ Extremely □ |

| **MODULE 13: Health-related QOL - EQ-5D-5L** | | | |
| --- | --- | --- | --- |
| Under each heading, please cross the ONE box that best describes your health TODAY | | | |
| **231** | *MOB* | Mobility  0 = I have no problems in walking about  1 = I have slight problems in walking about  2 = I have moderate problems in walking about  3 = I have severe problems in walking about  4 = I am unable to walk about | □ |
| **232** | *SCAR* | Self-care  0 = I have no problems washing or dressing myself  1 = I have slight problems washing or dressing myself  2 = I have moderate problems washing or dressing myself  3 = I have severe problems washing or dressing myself  4 = I am unable to wash or dress myself | □ |
| **233** | *UAC* | Usual activities (e.g. work, study, housework, family or leisure activities)  0 = I have no problems doing my usual activities  1 = I have slight problems doing my usual activities  2 = I have moderate problems doing my usual activities  3 = I have severe problems doing my usual activities  4 = I was unable to do my usual activities | □ |
| **234** | *PAIN* | Pain/ discomfort  0 = I have no pain or discomfort  1 = I have slight pain or discomfort  2 = I have moderate pain or discomfort  3 = I have severe pain or discomfort  4 = I have extreme pain or discomfort | □ |
| **235** | *AXDP* | Anxiety/ depression  0 = I am not anxious or depressed  1 = I am slightly anxious or depressed  2 = I am moderately anxious or depressed  3 = I am severely anxious or depressed  4 = I am extremely anxious or depressed | □ |
| **236** | *GSCA* | We would like to know how good or bad your health is TODAY. This scale is numbered from 0 to 100. 100 means the best health you can imagine. 0 means the worst health you can imagine. Indicate where on the scale your health is TODAY. 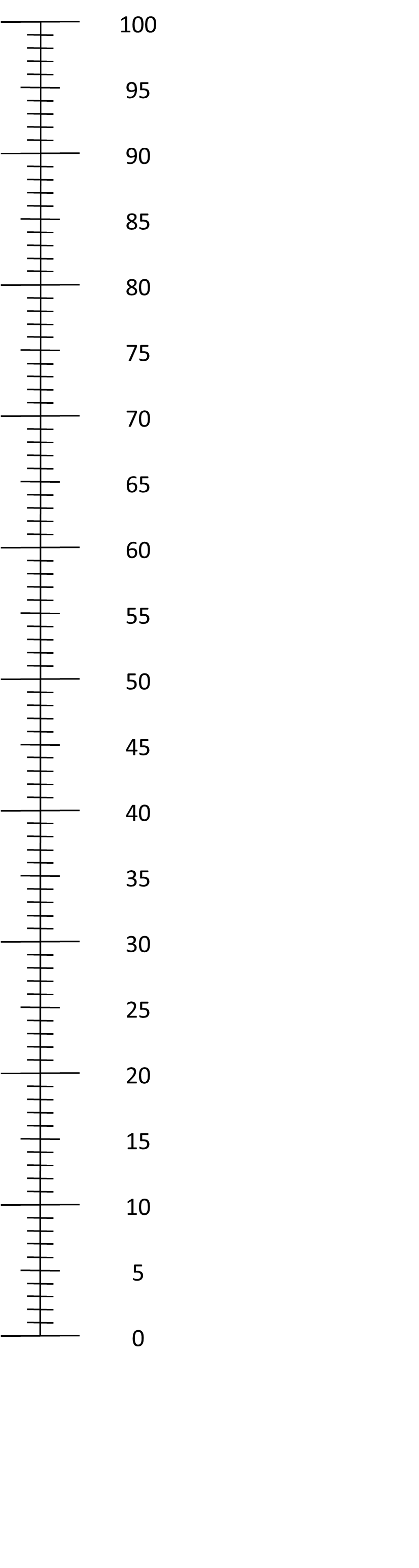 The best health you can imagine  The worst health you can imagine | Score □□ |

| **MODULE 14: Women’s Health Section** | | | | | |
| --- | --- | --- | --- | --- | --- |
| **237** | *MEN* | How old were you when your periods started? | | | □ □ years  Don’t know □ |
| **238** | *NPR* | How many times have you been pregnant? | | | □ □ |
| **239** | *BOR* | How many babies have you given birth to? | | | □ □ |
| **240** | *PRE* | Are you currently pregnant? | | | Yes □ No □ Don’t know □  If yes, end module |
| **241** | *HYS* | Have you had your womb removed (known as a hysterectomy) | | | Yes □ No □ Don’t know □ |
| **242** | *DRE* | IF YES, when? __________________ | | | □ □ /□ □ /□ □ □ □ |
| **243** | *OVR* | IF YES, were your ovaries removed at the time? | | | Yes □ No □ Don’t know □ |
| **244** | *REG* | Do you have regular periods; **defined as occurring every**  **occurring every 21 to 35 days?** | | | Yes □ No □  If no, skip to 246 |
| **245** | *DPE* | IF YES, when did you have your last period? | | | □ □ /□ □ /□ □ □ □  Complete, then skip to 248 |
| **246** | *DLM* | IF NO, when was your last period? | | 3 months ago □ 6 months ago □ 1 year ago □ more than 1 year□ | |
| **247** | *DRO* | For how long have you not had even a drop of blood? | | 3 months ago □ 6 months ago □ 1 year ago □ more than 1 year□ | |
| **248** | *CON* | Are you on/using a contraceptive? | | | Yes □ No □  If no, skip to 250 |
| **249** | *WCO* | IF YES which one? | Oral contraceptive pill □ IUD □  Injectables (known as depo) □  Condom □ | | |
| **250** | *HRT* | Are you on hormone replacement therapy (hormone therapy) | | | Yes □ No □ |
| **251** | *HTY* | IF YES, what type? | | | ________________________ |
| **252** | *STE* | Have you been sterilized (i.e. your tubes have been tied)? | | | Yes □ No □ Don’t know □ |

Thank you!
